# Common Data Elements for Workforce and Staffing Measurements in Long-Term Care

**DOI:** 10.1093/geroni/igab046.553

**Published:** 2021-12-17

**Authors:** Charlene Chu, Franziska Zúñiga, Kirsten Corazzini

## Abstract

The workforce in residential long-term care (LTC) is key in providing high-quality, person-centered care for residents. However, low staffing and adverse staffing outcomes such as turnover or job dissatisfaction hinder the provision of high-quality care. International research can add valuable insights for policy and practice by learning from different settings and cultures. The initiative “To Harmonize Research In long-term care liVing Environments (WE-THRIVE)”, is led by an international group of LTC researchers to identify common data elements (CDE) for cross-comparative research that support older adults thriving in LTC. In this symposium, we will present an overview of the WE-THRIVE initiative with a specific focus on CDEs and measurement. The first talk will provide the context for the WE-THRIVE initiative, and discuss the collaborative and iterative processes required to develop the initial CDEs in the area of workforce and staffing. In the second talk, we will discuss which staff should be “in the house” to meet the needs of residents during and after a pandemic, and what type of workforce data system should be available to ensure the best quality outcomes for residents and carers. Next, current issues in the measurement of staffing in LTC based on a review of reviews of staffing’s relationship to quality of care will be discussed. Finally, we extend the debate to consider theoretical and empirical explanations for the relationship between staffing and quality in LTC and the promotion of person-centred care outcomes.

